# A dataset of Indonesian tweets and daily market data for non-blue-chip stocks in the Indonesia Stock Exchange

**DOI:** 10.1016/j.dib.2026.113338

**Published:** 2026-10-08

**Authors:** Michael Sanjaya, Kuncahyo Setyo Nugroho, Matthew Martianus Henry, Vera Intanie Dewi, Bens Pardamean

**Affiliations:** a Computer Science Department, BINUS Graduate Program, Master of Computer Science, Bina Nusantara University, Jakarta, 11530, Indonesia; b Bioinformatics and Data Science Research Center, Bina Nusantara University, Jakarta, 11530, Indonesia; c Faculty of Economics and Business, Padjadjaran University, Bandung, 40115, Indonesia

**Keywords:** Indonesian stock market, Social media tweets, Market data, Sentiment analysis, Stock price forecasting

## Abstract

This paper describes a dataset of Indonesian tweets from X (formerly Twitter) and daily market data of 15 non-blue-chip stocks from the Indonesia Stock Exchange. The collection period is from 1 January 2022 to 31 December 2024. A total of 8968 tweets were collected using ticker-specific keyword queries through the X API, and 7672 tweets remained after preprocessing. The corresponding market data consists of 10,830 daily records downloaded from Yahoo Finance, including open, high, low, and close prices, and trading volume. The tweet texts have been cleaned of noise, and emojis have been replaced with descriptive words to preserve emotional context. Each tweet was labelled independently by three annotators as positive, negative, neutral, or irrelevant based on written guidelines, and the final label was determined by majority voting. Agreement reached a Fleiss' Kappa of 0.8811, and the final labels consist of 3490 neutral, 1753 positive, 1751 irrelevant, and 678 negative tweets. The dataset is available in six separate CSV files that share a common ticker column: the Indonesian Tweet Data, the English-translated Tweet Data, the raw Market Data, the annotator labels, the stock screening values, and the Combined Data, which integrates market data and tweet data and contains 16,395 calendar-aligned daily records, of which 5565 are forward-filled and marked by an indicator column. This dataset is useful for research on social media sentiment analysis, short-term stock price prediction, and market euphoria prediction in the Indonesian capital market.

Specifications Table

**Table:** 

Subject	Computer Sciences
Specific subject area	Natural Language Processing, Time Series Analysis
Type of data	Tabular data (CSV files)
Data collection	The daily market data for 15 non-blue-chip stocks were downloaded from Yahoo Finance using the yfinance Python library, and the quarter-end price to earnings ratios of the selected stocks were taken from Stockbit. Tweet data were retrieved from X (formerly Twitter) using the X API v2 (endpoint of GET /2/tweets/search/all) on 15 April 2026 by running targeted keyword queries for each stock ticker. The data collection period is from 1 January 2022 to 31 December 2024. The tweet texts were cleaned, anonymised and normalised. Each cleaned tweet was labelled as positive, negative, neutral, or irrelevant by three annotators, and agreement was measured with Cohen’s Kappa and Fleiss’ Kappa.
Data source location	The tweets were collected at Bina Nusantara University, Indonesia.
Data accessibility	Repository name: Mendeley Data Data identification number: 10.17632/rkpkkd2mhf.5 Direct URL to data: https://data.mendeley.com/datasets/rkpkkd2mhf/5 Instructions for accessing these data: The dataset is publicly available for research purposes. No login is required to download.
Related research article	None

## 1 Value of the Data

• To the best of our knowledge, this is the first publicly available dataset that connects Indonesian-language tweets on X (formerly Twitter) with daily market data (open, high, low, close prices, and trading volume) for 15 non-blue-chip stocks on the Indonesia Stock Exchange (IDX) [1]. The dataset contains 7672 tweets with human-annotated sentiment labels and 10,830 daily market records collected over three years (2022 to 2024). In addition, a combined file containing 16,395 calendar-aligned daily records is provided for multimodal analysis, and the 5565 rows in it that were produced by forward filling are marked by an indicator column. One closely related Indonesian resource is ID-SMSA [2], which provides 3288 tweets on the ten largest stocks by market capitalisation, with three sentiment classes and without market data. This dataset addresses the gap left by most existing financial datasets, which primarily focus on English-language text or large-cap stocks, by covering non-blue-chip stocks, by adding an irrelevant class, and by pairing every daily tweet metric with daily market data for the Indonesian market.
• This dataset is valuable for research focusing on the Indonesian stock market, which is characterized by high retail participation and particular speculative dynamics [3]. The tweet data contains informal Indonesian financial language and slang commonly used in social media discussions, providing a useful resource for sentiment analysis and research on market euphoria [4].
• The components of the dataset can be individually reused for different research purposes. The tweet data is useful for supervised natural language processing tasks such as sentiment classification and the benchmarking of Indonesian language models on informal financial text. The daily market data can be used individually to analyze prices and trading volume, as well as for time-series forecasting [5].
• The combined daily aggregated tweet metrics and daily market data allow for multimodal machine learning research by integrating social media-derived features with financial time-series data. It is designed to integrate easily with other financial databases that use explicit merge keys, such as stock tickers and dates.
• This dataset provides a foundation for investigating herd behavior and speculative price movements in non-blue-chip stocks. Researchers can reuse the paired data to study the relationship between social media activity, sentiment, and short-term market volatility in emerging markets [6].

## 2 Background

Retail investors in the Indonesian capital market often discuss stock movements on social media platforms, such as X (formerly Twitter). These discussions can influence trading volumes and price volatility, especially for non-blue-chip companies that experience high levels of retail participation and speculative trading [3]. The number of retail investors in Indonesia reached 14.87 million in December 2024 [7]. This substantial level of retail participation may contribute to market characteristics that differ from those observed in developed markets.

Public datasets of Indonesian tweets along with daily market data are limited. Most available tweet datasets are in English and cover only developed markets or large-cap stocks [3]. One closely related Indonesian resource is ID-SMSA [2], which contains 3288 tweets discussing the ten largest stocks by market capitalisation on the IDX as of March 2023, collected between 12 January 2021 and 1 March 2024 and labelled as positive, neutral, or negative by two annotators, with a Cohen’s Kappa of 0.779.

Work that links text sentiment to price movement is more developed for markets outside Indonesia. Architectures that combine a transformer encoder for text with a recurrent network for the price series have been reported for English-language news and social media [8], a systematic review covers a broad set of news-based prediction studies [3], and social media discussion volume has been reported to move ahead of trading volume in developed markets [5]. All of this work depends on text and market records that are already paired, and that pairing is what public Indonesian resources currently lack.

There are three differences between ID-SMSA and the dataset presented here. First, the stock universe differs because ID-SMSA covers the ten largest-capitalisation stocks, whereas this dataset covers 15 non-blue-chip stocks selected through a screening procedure based on speculative price and volume behaviour. Second, the label set also differs, because ID-SMSA uses three sentiment classes, while this dataset adds a fourth class, irrelevant, so that posts returned by a ticker query without any view on the stock can be separated from the sentiment classes. Third, the scope differs, because ID-SMSA provides tweets only, while this dataset pairs the tweets with 10,830 daily market records and provides a merged file of 16,395 calendar-aligned daily rows in which each daily tweet metric is aligned to a trading date through explicit ticker and date keys.

To fill this gap, this dataset pairs Indonesian tweets with daily market data for 15 non-blue-chip stocks from the IDX. The 15 stocks were screened using two technical criteria, the 14-day Relative Strength Index (RSI) and the volume ratio, and the price-to-earnings (P/E) ratio of each selected stock is reported as a supporting indicator of valuation, as described in Section 4.1. Tweet data were collected using the X API, while market data were retrieved using yfinance, covering the period from 1 January 2022 to 31 December 2024. The data was then carefully cleaned and anonymised to protect privacy.

This data article makes three contributions. First, a manually annotated Indonesian sentiment resource is released for 15 IDX stocks selected for speculative price and volume behaviour, with a fourth class that separates posts carrying no position on the stock from the three sentiment classes. Second, every daily tweet metric is paired with a daily market record for the same ticker through explicit ticker and date keys, so the text side and the price side can be used together without a separate matching step. Third, the alignment decisions that affect downstream forecasting are recorded as columns inside the released files, namely the time zone of the posting time, the trading session assigned to each tweet, and the flag that separates observed market rows from imputed ones.

This article is organised as follows. Section 3 describes the released files, their attributes, and the descriptive characteristics of the data. Section 4 describes the construction procedure, covering stock selection, market data collection and imputation, tweet collection and preprocessing, manual annotation, daily aggregation, and merging. The Limitations section states the conditions under which the data should be interpreted, and the Ethics Statement records the terms under which the data were collected and released.

## 3 Data Description

The dataset consists of pairs of Indonesian tweet data and daily market data of 15 non-blue-chip stocks listed on the Indonesia Stock Exchange (IDX). The 15 stocks were selected through the quantitative screening described in Section 4.1. All 15 stocks recorded at least one trading day on which the 14-day Relative Strength Index was above 70 and the volume ratio was above 2.0 at the same time. On those days, the price to earnings ratio was also checked as a supporting indicator of valuation. Strong buying pressure together with unusually high trading activity is the pattern associated with speculative trading [6], and where the price to earnings ratio is also high on the same days, the price has moved ahead of the reported earnings. This makes the selected stocks suitable for studying sentiment and price movements in stocks exhibiting potentially speculative market characteristics. The 15 stocks are:

• KARW (PT Meratus Jasa Prima Tbk).
• FORU (PT Fortune Indonesia Tbk).
• SRAJ (PT Sejahteraraya Anugrahjaya Tbk).
• PANI (PT Pantai Indah Kapuk Dua Tbk).
• DSSA (PT Dian Swastatika Tbk).
• SGER (PT Sumber Global Energy Tbk).
• TPIA (PT Chandra Asri Petrochemicals Tbk).
• BRMS (PT Bumi Resources Minerals Tbk).
• MLPT (PT Multipolar Technology Tbk).
• BRPT (PT Barito Pacific Tbk).
• TOBA (PT TBS Energi Utama Tbk).
• AUTO (PT Astra Otoparts Tbk).
• IMAS (PT Indomobil Sukses Internasional Tbk).
• PSAB (PT J Resources Asia Pasifik Tbk).
• KONI (PT Perdana Bangun Pusaka Tbk).

This dataset is publicly available in the Mendeley Data Repository [1] and consists of six separate files: the Indonesian Tweet Data (Tweet Data - ID.csv), the English translated Tweet Data (Tweet Data - EN.csv), the Market Data (Stock Market Data.csv), the Combined Data (Combined Data.csv), the annotation matrix (Annotation Matrix.csv), and the screening values of the selected stocks (Stock Screening Values.csv). The repository also contains a README file that describes the content of each file and lists its columns, and a Jupyter notebook, Dataset Construction.ipynb, that downloads the market data with yfinance and repeats the forward filling, technical feature calculation, daily aggregation, merging, and stock screening described in Section 4. The Combined Data is a merged file obtained by performing a left join between the forward filled market series described in Section 4.3 and the daily aggregated tweet metrics, on the common columns ticker and date. This file also includes the technical features (rsi_14, price_change_pct, and volume_change_pct) calculated from the Market Data. The annotation matrix holds the label assigned by each of the three annotators for every tweet together with the final label, and is described in Section 4.6. The screening values file holds, for each selected stock, the number of trading days on which both technical conditions described in Section 4.1 were met, the first and last of those dates, the highest value reached by each technical indicator, and the price to earnings ratio on those days.

The Tweet Data (filename: Tweet Data - ID.csv) consists of 7672 rows of Indonesian tweets that were gathered by targeted keyword queries on X (formerly Twitter) using the X API. Each row represents a single tweet and contains the stock ticker, the full tweet text (full_text), the date and time of posting (created_at), the trading session to which the tweet is assigned (trading_session_date), engagement metrics (favorite_count, retweet_count, reply_count, and is_quote_status), and the sentiment label assigned during the annotation stage, as detailed in Table 1. An English translated version of this file is also provided as Tweet Data - EN.csv. The translation was produced with the document translation feature of Google Translate (translate.google.com), with the source language set to Indonesian and the target language set to English. The cleaned tweet texts were exported into a document, submitted to the service as a single batch, and written back into the spreadsheet in the original row order. Google Translate is a hosted service and does not expose a public version number, so the access date is reported in place of a version. The translation was carried out on 1 May 2026. The translated output was then reviewed against the Indonesian source, and manual correction was applied to 466 of the 7672 rows. Three kinds of translation problem were found. First, ticker symbols written in lower case in the source were treated as ordinary words, so the ticker was either translated or dropped and no longer appeared in the English text. Second, some rows were returned untranslated or translated only in part. Third, stock trading slang was rendered by its literal dictionary meaning, for example “*gorengan*”, which denotes a speculative and heavily traded stock, was returned as “fried food”, “*bandar*”, which denotes the large participant moving the price, was returned as “bookie”, and “*barang*”, which denotes the shares themselves, was returned as “goods”. Translation was applied after the cleaning stage, so Tweet Data - EN.csv contains the same 7672 rows, the same metadata columns, and the same sentiment labels as Tweet Data - ID.csv. The sentiment labels were assigned on the Indonesian text and were not reassigned on the translated text, so the English file is provided for readability and is not a separately annotated resource. All tweet texts have been anonymized and cleaned, including usernames, user mentions, external URLs, and personal identifiers, to protect user privacy.

**Table 1:** Description of attributes in the tweet data.

Attribute	Data Type	Description
ticker	String	Stock ticker symbol. Used as the primary key for merging with market data
full_text	String	Cleaned and anonymised full text of the tweet
created_at	Datetime	Date and time the tweet was posted in Western Indonesia Time (UTC+7)
trading_session_date	Datetime	Date of the IDX trading session to which the tweet is assigned, as defined in Section 4.7. Left empty for tweets posted after the last trading session of the observation period
favorite_count	Integer	Engagement metric indicating number of times the tweet has been liked/favorited
retweet_count	Integer	Engagement metric indicating number of times the tweet has been retweeted
reply_count	Integer	Engagement metric indicating number of replies the tweet has received
is_quote_status	Boolean	Boolean flag indicating whether the tweet is a quote tweet (true/false). This is used for observing the dynamics of quotes in stock discussions
sentiment	String	Sentiment label of the tweet, recorded as Positive, Negative, Neutral, or Irrelevant

The Market Data (filename: Stock Market Data.csv) contains 10,830 rows of raw daily OHLCV (open, high, low, close prices, and trading volume) data for the 15 selected stocks. Attributes are described in Table 2. The data was sourced from Yahoo Finance through the yfinance Python library. This file holds exchange trading days only and is released without imputation, so weekends and public holidays are absent from it. The forward filled version of the series, which expands to 16,395 daily rows, is provided inside Combined Data.csv and is described in Section 4.3.

**Table 2:** Description of attributes in the market data.

Attribute	Data Type	Description
ticker	String	Stock ticker symbol
company_name	String	Full company name
date	Datetime	Trading date
open	Float	Opening price of the stock on that trading day
high	Float	Highest price reached during the trading day
low	Float	Lowest price reached during the trading day
close	Float	Closing price of the stock on that trading day
volume	Integer	Total number of shares traded during the day

In addition, a merged version named Combined Data.csv is provided that contains 16,395 rows. The row count follows directly from the calendar expansion described in Section 4.3, because the observed series runs from 3 January 2022 to 30 December 2024, which covers 1093 calendar days, and 1093 days for each of the 15 tickers give 16,395 rows. This file combines the daily market data with daily aggregated tweet metrics with a left join on the common columns ticker and date. It consists of all attributes from both datasets as well as the technical features calculated. The attributes are described in Table 3.

**Table 3:** Description of attributes in the combined data.

Attribute	Data Type	Description
ticker	String	Stock ticker symbol. Primary key for merging
date	Datetime	Calendar date, including weekends and public holidays
open	Float	Opening price of the stock on that trading day
high	Float	Highest price reached during the trading day
low	Float	Lowest price reached during the trading day
close	Float	Closing price of the stock on that trading day
volume	Integer	Total number of shares traded during the day
is_imputed	Integer	Set to 1 when the market values in the row were carried forward from the previous trading day, and set to 0 when the row is an observed trading day
tweet_count	Integer	Number of tweets for the ticker on that date, counted without the tweets labelled as irrelevant
daily_avg_score	Float	Average of the manual sentiment labels (−1.0 to +1.0) for the ticker on that date, calculated without the tweets labelled as irrelevant
rsi_14	Float	14-day Relative Strength Index calculated from the closing prices. Left empty for the first 14 days of each ticker, as described in Section 4.3
price_change_pct	Float	Daily price change percentage from the previous trading day
volume_change_pct	Float	Daily volume change percentage from the previous trading day

Fig. 1 shows the daily tweet count across all 15 tickers from January 2022 to December 2024. Fig. 2 presents an overview of the dataset characteristics, including (a) annual tweet counts from 2022 to 2024, (b) the top 10 most mentioned tickers, (c) the distributions of closing prices in IDR, and (d) distribution of daily trading volumes from the market dataset.

**Fig. 1:**
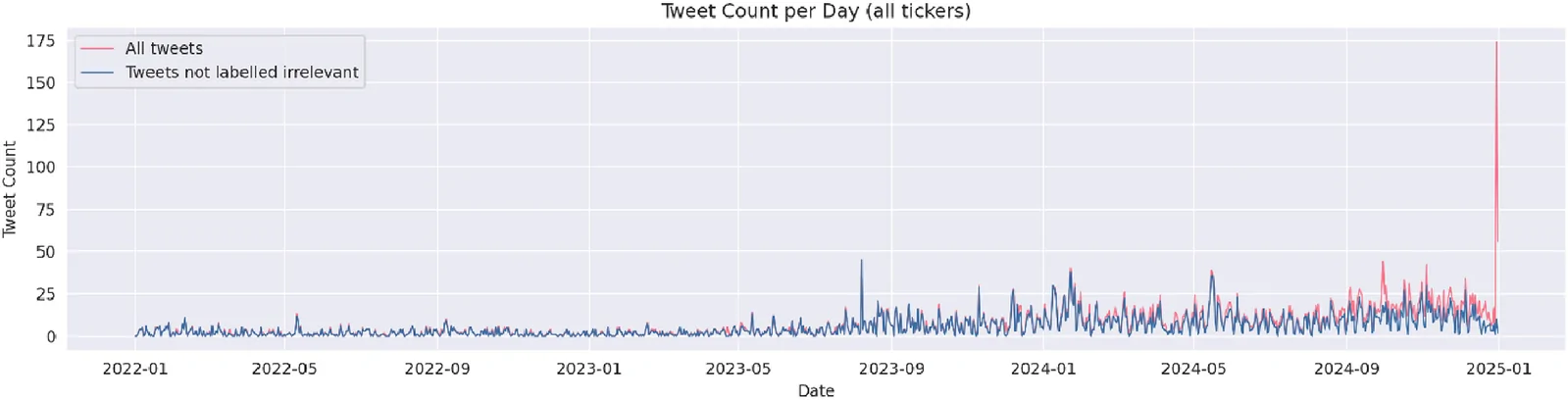
Daily number of tweets collected across all 15 tickers between January 2022 and December 2024.

**Fig. 2:**
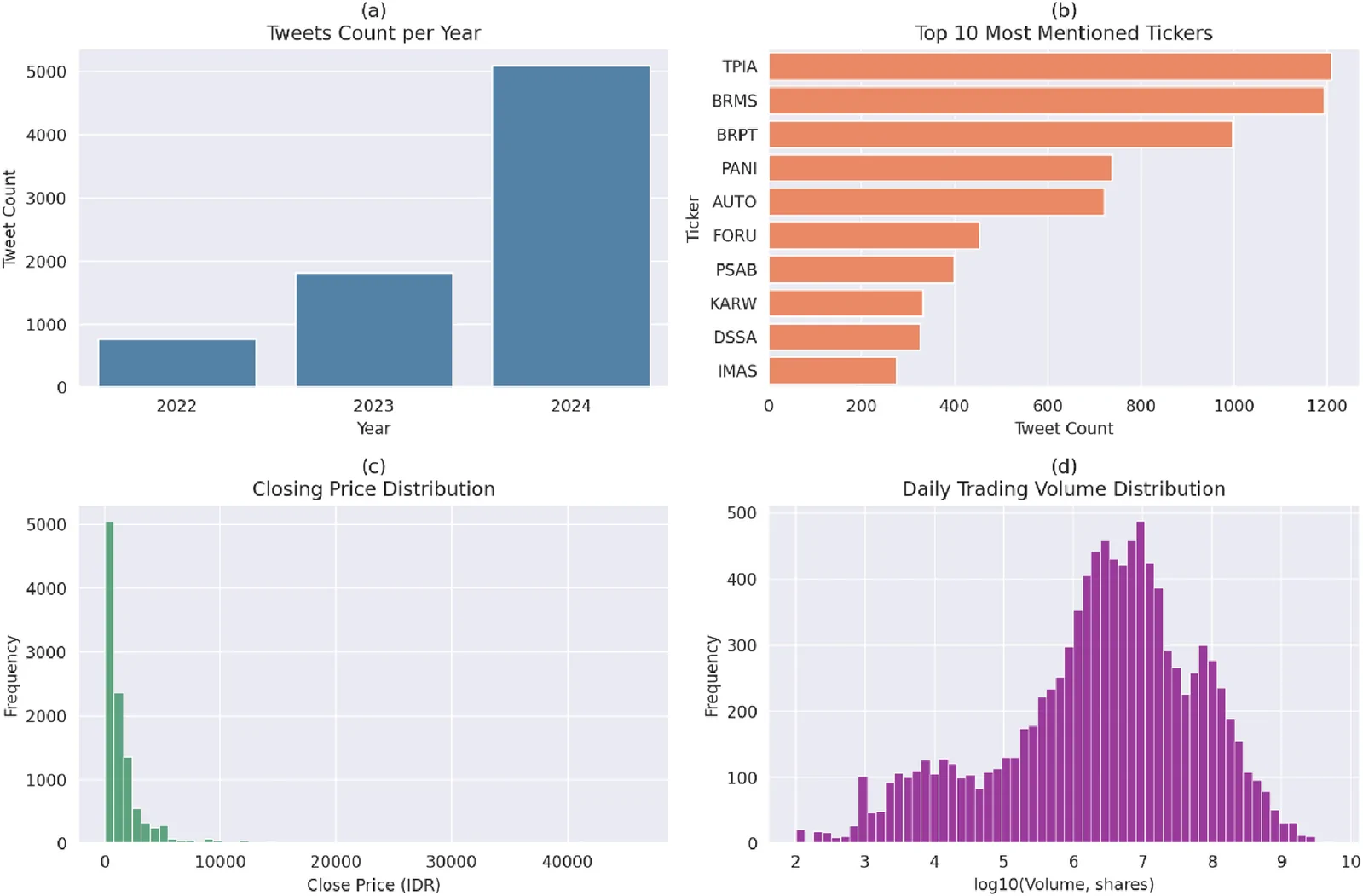
General information about the dataset (a) Annual tweet counts by year (2022–2024). (b) Most mentioned tickers within the top 10 by tweet count. (c) Market dataset closing price distribution (in IDR). (d) Market dataset daily trading volume distribution (log10 scale).

Annotation Matrix.csv contains 7672 rows, one for each tweet, in the same order as the rows of Tweet Data - ID.csv and Tweet Data - EN.csv. It holds the row number, the ticker, the label assigned by each of the three annotators, the final label, and the level of agreement among the three. The attributes of Annotation Matrix.csv are described in Table 4.

**Table 4:** Description of attributes in the annotation matrix.

Attribute	Data Type	Description
row	Integer	Position of the tweet in Tweet Data - ID.csv and Tweet Data - EN.csv, counted from 1 for the first data row
ticker	String	Stock ticker symbol, identical to the ticker of the same row in the Tweet Data files
a1	String	Label assigned by annotator A1, recorded as Positive, Negative, Neutral, or Irrelevant
a2	String	Label assigned by annotator A2, recorded with the same four categories
a3	String	Label assigned by annotator A3, recorded with the same four categories
final_label	String	Label decided by majority voting, or by the final review described in Section 4.6 when the three labels differ. Identical to the sentiment column of the Tweet Data files
agreement	Integer	Number of annotators who assigned the most frequent label: 3 when all three agree, 2 when two agree, and 1 when the three labels differ

Stock Screening Values.csv contains 15 rows, one for each selected stock. It holds the ticker, the company name, the number of days on which both technical conditions described in Section 4.1 were met on the same day, the first and last of those dates, the highest value reached by each technical indicator on those days together with the date on which it occurred, the number of those days on which the price to earnings ratio was above 25, and the highest price to earnings ratio among them. The attributes of Stock Screening Values.csv are described in Table 5.

**Table 5:** Description of attributes in the Stock Screening Values.

Attribute	Data Type	Description
ticker	String	Stock ticker symbol
company_name	String	Full company name
qualifying_days	Integer	Number of trading days on which the 14-day Relative Strength Index was above 70 and the volume ratio was above 2.0 on the same day. A day that meets only one of the two conditions is not counted
first_qualifying_date	Datetime	Earliest date on which both conditions in qualifying_days were met
last_qualifying_date	Datetime	Latest date on which both conditions in qualifying_days were met
max_rsi_14	Float	Highest 14-day Relative Strength Index among the qualifying days, calculated with Eqs. (3) to (7)
max_rsi_14_date	Datetime	Date on which max_rsi_14 occurred
max_volume_ratio	Float	Highest volume ratio among the qualifying days. The volume ratio is the volume of the day divided by the mean volume of the 20 calendar days ending on that day, with each non-trading day carrying the volume of the previous trading day
max_volume_ratio_date	Datetime	Date on which max_volume_ratio occurred
qualifying_days_pe_above_25	Integer	Number of qualifying days on which the price to earnings ratio was above 25. A day on which the company had zero or negative earnings over the latest four quarters has no ratio and is not counted
max_pe_qualifying	Float	Highest price to earnings ratio among the qualifying days. Very large values occur when the earnings of the latest four quarters are close to zero

## 4 Experimental Design, Materials and Methods

The dataset construction begins with stock selection, market data collection, tweet data collection, preprocessing of tweet data, sentiment scoring, and the integration of the collected data. The overview of the workflow is shown in Fig. 3.

**Fig. 3:**
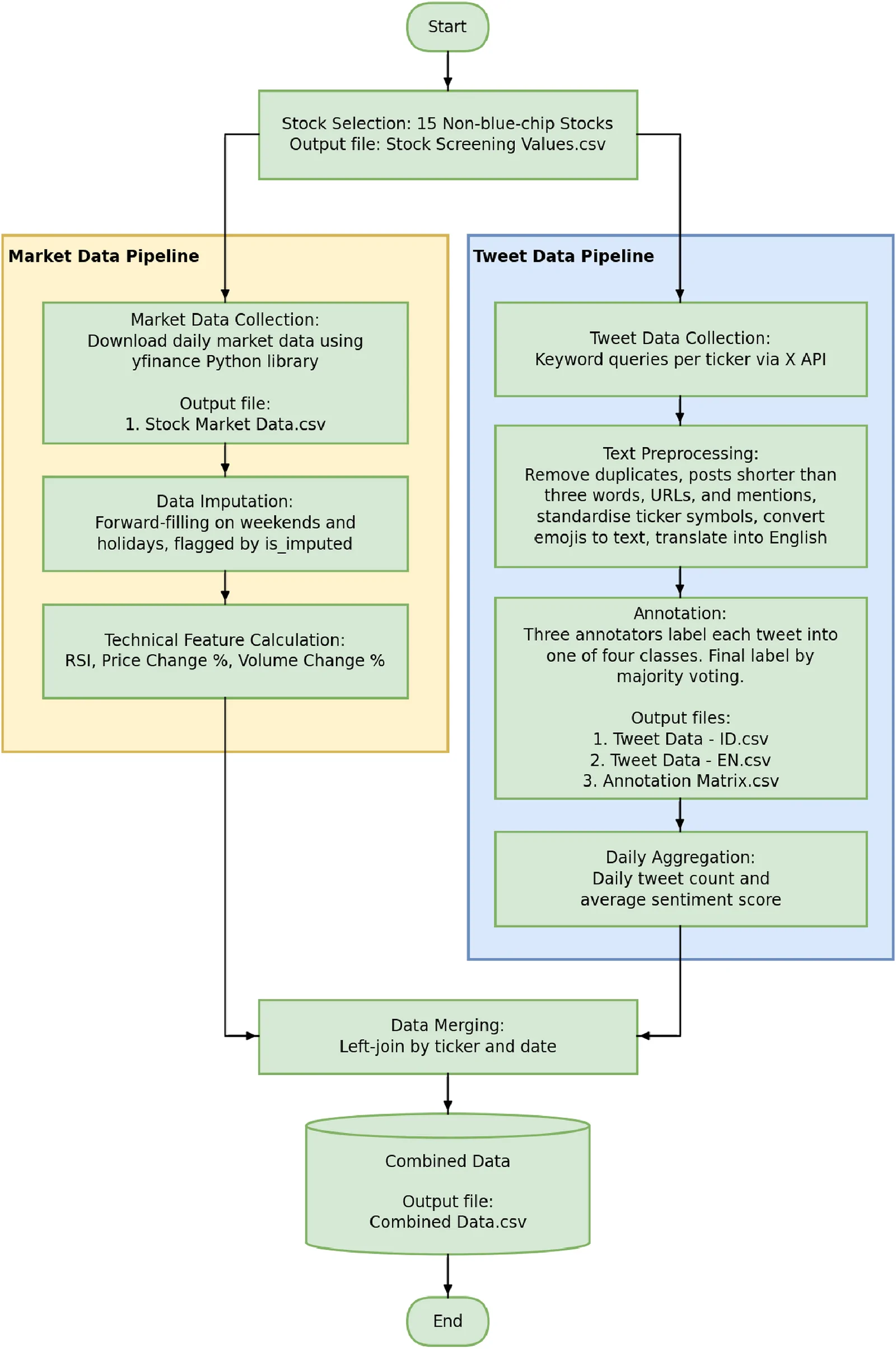
Overview of the workflow for the construction of the dataset.

### 4.1 Stock selection

The 15 non-blue-chip stocks were selected based on their previous indication of market speculation activity. The term non-blue-chip is used in this article as a working label for the stocks that the screening procedure below selects. It is not based on a classification issued by the Exchange. These stocks were assessed using a combination of two technical filters and one fundamental indicator. A stock was selected when the two technical filters were met on the same trading day at least once. Both filters use only daily prices and trading volumes, so they can be applied to every stock listed on the Exchange. The fundamental indicator was then calculated for the selected stocks on the days that met both technical filters, as a supporting indicator instead of a selection condition, and it shows for each selected stock whether its strong momentum and high trading volume occurred at a valuation that its reported earnings do not support.

The first technical filter is the 14-day Relative Strength Index (RSI), where a value above 70 would suggest that the stock is in the overbought status, and buying pressure is made dominant in the market [9]. The second technical filter is the volume ratio, which is calculated by taking the current daily trading volume and comparing it to the 20-day simple moving average, with the threshold set above 2.0. A volume ratio that is above 2.0 suggests that the current volume is more than double the average volume over the past 20 days, indicating higher than normal trading activity [10]. The fundamental indicator is the Price to Earnings (P/E) Ratio, with a reference level of 25 to identify stocks that are priced higher relative to their reported earnings. The higher levels suggest that market speculation is driving the stock to be overvalued. The two technical thresholds select stocks whose price and volume behaviour departs from their own recent norm. The Indonesia Stock Exchange defines Unusual Market Activity (UMA) as unusual trading activity or price movement of a security within a certain period which, in the assessment of the Exchange, has the potential to disrupt orderly, fair, and efficient trading [11]. The screening applied here follows the same idea of a departure from normal price and volume behaviour [12], but the three threshold values were set for this study and are not the criteria used by the Exchange. The number of days on which both technical conditions were met, the first and last of those dates, and the highest value of each indicator are reported for every selected stock in Stock Screening Values.csv. The price to earnings ratio on each qualifying day was taken from Stockbit (stockbit.com), an Indonesian online stock investment platform that reports the trailing twelve-month ratio of each listed company from its financial statements. The ratio at the end of the latest quarter available on that day was carried forward with the daily closing price from Yahoo Finance, and a quarter was used from three months after its end, which is the latest reporting deadline under OJK Regulation No 14/POJK.04/2022 [13]. The values were accessed on 15 April 2026 and are listed in Dataset Construction.ipynb. The number of qualifying days on which this ratio was above 25 and its highest value on a qualifying day are also reported in Stock Screening Values.csv.

### 4.2 Market data collection

After stock selection, daily market data were obtained from Yahoo Finance. The data were extracted using the yfinance Python library (version 0.2.66) running on Python 3.13. The market variables obtained are open, high, low, close prices, and trading volume (OHLCV) for the period 1 January 2022 to 31 December 2024.

The extraction process retrieved individual historical records for each of the 15 selected stock tickers. These raw records initially reflected only exchange trading days. Therefore, the raw data initially contained missing data points due to weekends or public holidays.

### 4.3 Market data imputation and technical feature calculation

Data imputation was performed after the raw market data had been collected. These gaps from raw market data were filled by forward-filling to obtain a continuous time series without leaving any empty rows. Forward-filling was chosen because the last traded price stays in force until the next trading session opens. Trading volume was carried forward as well so that every row holds a complete set of columns, and every carried row is marked by the is_imputed column. This method transfers data from the last trading day onward until the next trading day. The imputation process was applied to all tickers to prevent cross-stock data mixing. The final market data expanded from 10,830 observed daily rows to 16,395 rows, because the 722 trading days between 3 January 2022 and 30 December 2024 span 1093 calendar days, and 1093 days for each of the 15 tickers give 16,395 rows. Of these, 5565 rows (33.94%) are filled rows. Each ticker was forward-filled individually to address the calendar dates caused by weekends and public holidays.

Finally, technical feature calculations were performed on the continuous OHLCV data. This process is important for removing price-scale differences across different stock tickers. The daily price change percentage is the simple percentage change between two consecutive closing prices, and it is determined by measuring the relative difference between the current day’s close price and the previous day’s close price, as shown in Eq. (1) [14].

(1) $$Price\;Change\;Percentage=\frac{Close_{t}-Close_{t-1}}{Close_{t-1}}\times 100$$

Meanwhile, the daily volume change percentage measures the relative difference in trading volume as shown in Eq. (2). On days affected by forward-filling where prices and volumes do not change, both percentage change values become zero. The same value of zero is recorded on the first day of each ticker, which has no previous day, and for volume_change_pct on a day that follows a day with zero volume, for which Eq. (2) has no defined value.

(2) $$Volume\;Change\;Percentage=\frac{Volume_{t}-Volume_{t-1}}{Volume_{t-1}}\times 100$$

The 14-day Relative Strength Index (RSI) was built as a momentum indicator to measure the magnitude and speed of recent price movements. The RSI converts raw closing prices into a normalized number between 0 and 100, therefore comparisons can be made between companies at different price levels. The RSI computation follows the original formulation of Wilder [15] and starts with the daily price difference between the current closing price and the previous day’s closing price as mentioned in Eq. (3).

(3) $$\Delta Price_{t}=Close_{t}-Close_{t-1}$$

If the daily price difference is positive, the value is categorized as a gain; otherwise, it is categorized as a loss as shown in Eq. (4) [15]. Gains and losses are separated because RSI measures the relative strength of upward and downward price movements.

$$\text{Gain}=\left\{ \begin{array}{c} \Delta Price_{t},\;if\Delta Price_{t}>0\\ 0,\;otherwise \end{array}\right.$$

(4) $$\begin{array}{c} \mathrm{Loss}=\left\{ \begin{array}{c} |{\Delta \mathrm{Price}}_{t}|,\;\mathrm{if}\Delta \mathrm{Pric}e_{t}<0\\ 0,\;\mathrm{otherwise} \end{array}\right. \end{array}$$

After daily gains and losses were calculated, the first average gain and first average loss were calculated by the arithmetic mean across the first 14 periods of the forward-filled series as shown in Eq. (5) [15]. Because 14 price differences are needed before the first average exists, rsi_14 is left empty for the first 14 days of each ticker, from 3 to 16 January 2022.

$$First\;AverageGain=\frac{\sum_{i=1}^{14}Gain_{i}}{14}$$

(5) $$First\;AverageLoss=\frac{\sum_{i=1}^{14}Loss_{i}}{14}$$

The average gain and loss were updated for the following periods using Wilder’s smoothing method [15], where the previous average values were combined with the current day’s gain or loss as shown in Eq. (6). $Average\;Gain_{t-1}$ and $Average\;Loss_{t-1}$ represent the average gain and loss from the previous trading day, while $Gain_{t}$ and $Loss_{t}$ represent the current day’s gain and loss values.

$$AverageGain_{t}=\frac{\left(AverageGain_{t-1}\times 13\right)+Gain_{t}\;}{14}$$

(6) $$AverageLoss_{t}=\frac{\left(AverageLoss_{t-1}\times 13\right)+Loss_{t}\;}{14}$$

The Relative Strength Index (RSI) value was then calculated using the ratio between the average gain and average loss as shown in Eq. (7) [15].

(7) $$RSI_{t}=100-\left[\frac{100}{1+\frac{AverageGain_{t}}{AverageLoss_{t}}}\right]$$

This stage provides a standardized daily measure of market velocity and volatility for each asset in the dataset. For example, in the case of AUTO, the price_change_pct and volume_change_pct are both 0.00 on 25, 26, 28 and 29 December 2024 as the values are carried forward from the previous day as shown in Table 6, Table 7. Then, these three technical features (rsi_14, price_change_pct, and volume_change_pct) were added as extra columns to the dataset.

**Table 6:** Raw market data before data imputation and technical feature calculation.

date	ticker	open	high	low	close	volume
2024-12-24	AUTO	2092.175	2101.351	2018.765	2046.294	1,016,200
2024-12-27	AUTO	2046.294	2055.470	2037.118	2037.118	530,900
2024-12-30	AUTO	2046.294	2110.527	2027.941	2110.527	2,132,400

**Table 7:** Market data after data imputation and technical feature calculation.

date	ticker	open	high	low	close	volume	rsi_14	price_change_pct	volume_change_pct
2024-12-24	AUTO	2092.175	2101.351	2018.765	2046.294	1,016,200	42.760	−2.193	−67.274
2024-12-25	AUTO	2092.175	2101.351	2018.765	2046.294	1,016,200	42.760	0.000	0.000
2024-12-26	AUTO	2092.175	2101.351	2018.765	2046.294	1,016,200	42.760	0.000	0.000
2024-12-27	AUTO	2046.294	2055.470	2037.118	2037.118	530,900	41.521	−0.448	−47.756
2024-12-28	AUTO	2046.294	2055.470	2037.118	2037.118	530,900	41.521	0.000	0.000
2024-12-29	AUTO	2046.294	2055.470	2037.118	2037.118	530,900	41.521	0.000	0.000
2024-12-30	AUTO	2046.294	2110.527	2027.941	2110.527	2,132,400	54.650	3.604	301.658

### 4.4 Tweet data collection

The tweet data were collected from X (formerly Twitter) using the X API v2, and the retrieval was carried out on 15 April 2026. Targeted queries were built for each of the 15 selected stocks. These queries used a combination of the unique ticker symbol and the full company name, joined by the OR operator (e.g., “$KARW” OR “PT Meratus Jasa Prima Tbk”). Only the ticker symbol and the company name change between the 15 queries, and both are listed in Section 3, so every query string can be reconstructed from this template. This search approach was used to capture conversations from different categories of investors on the platform.

The data collection covered the observation period from 1 January 2022 to 31 December 2024 to align with the historical market data, and the results of each query were collected page by page until no further posts were returned for that period. No additional filters (such as media type or quote status) were applied during collection, so the retrieved set is not restricted to Indonesian-language posts, and 8700 of the 8968 retrieved posts carry the Indonesian language code reported by the platform. The language of each post was judged by the annotators, and posts written in a language other than Indonesian were labelled as irrelevant, as described in Section 4.6. No record in the collected set carries a retweet prefix, so retweets are absent from the dataset, while quote posts are present and are marked by the is_quote_status field, which is true for 412 of the retrieved posts. The data collection process produced records containing both tweet text and associated metadata. The metadata included the posting time (created_at) and engagement metrics, including the numbers of favorites, retweets, and replies.

### 4.5 Tweet data preprocessing

The collected tweets were cleaned to remove noise and ensure user privacy. The first stage of preprocessing was removing duplicate tweets, tweets shorter than three words, external URLs, and user mentions. The three word threshold was set because the ticker symbol itself takes up one word in every retrieved post. After the ticker, the URL, and the user mention are removed, a post of fewer than three words leaves at most one content word, which is not enough context for an annotator to assign a sentiment class. Irrelevant non-alphanumeric characters and extra spaces were also removed to clean the text structure. After preprocessing, 7672 tweets remained from the 8968 tweets collected, and these form the released Tweet Data.

Data collection was performed separately for each of the 15 stock tickers using specific keyword queries on the X API. Because of this, a tweet that mentions more than one ticker (for example, “$KARW $PANI”) was retrieved once for every ticker whose query matched it. The duplicate records of such a tweet were removed in the preprocessing stage described above, and one record was kept. A tweet that mentions several of the selected tickers therefore appears once in the released files and is linked to a single ticker.

The text was then prepared for the annotation stage. Financial tickers were standardised by removing the dollar sign prefix (e.g., converting $KARW to KARW). The dollar sign was removed so that the ticker is kept as a single word in the released text, because special characters are often treated as noise during tokenisation by language models trained on this kind of data. In addition, emojis were translated to descriptive Indonesian text using a Python demoji library (version 2.0.0). For example, the emoji  was translated to “*tanda_silang*” (cross_mark). This step maintains the emotional context of the tweets. Table 8 shows a sample of tweet text before and after preprocessing. URLs in the raw tweets are masked as https://t.co/xxx to protect user privacy.

**Table 8:** Text cleaning sample comparison.

Raw Tweets	Cleaned Tweets
$KARW sekarang = $PANI 3 tahun yang lalu meratus line sedang proses merger & akuisisi dengan ad ports untuk mengelola pelabuhan patimban, dengan nilai transaksi -+ 30T https://t.co/xxx ***English translation:****$KARW is now $PANI 3 years ago, Meratus Line was in the process of merger and acquisition with Ad Ports to manage Patimban Port with a transaction value of 30T -+*https://t.co/xxx	KARW sekarang PANI 3 tahun yang lalu meratus line sedang proses merger amp akuisisi dengan ad ports untuk mengelola pelabuhan patimban dengan nilai transaksi 30T ***English translation:****KARW is now PANI 3 years ago Meratus Line was in the process of merger and acquisition with Ad Ports to manage Patimban Port with a transaction value of 30T*

Marah-marah karena kalah pilpres dan soal dinasti Marah-marah karena ketinggalan $KARW ARA terus2an dan dikandangin FCATiba2 4000 https://t.co/xxx ***English translation:** Angry because lost the presidential election and about the dynasty**Angry because he kept missing $KARW ARA and was caged by FCA**Suddenly 4000*https://t.co/xxx	Marah marah karena kalah pilpres dan soal dinasti tanda_silang Marah marah karena ketinggalan KARW ARA terus2an dan dikandangin FCA tanda_centang_putih_tebal Tiba2 4000 ***English translation:** Angry because lost the presidential election and about the dynasty cross_mark Angry because he kept missing KARW ARA and was caged by FCA check_mark_button Suddenly 4000*

### 4.6 Data annotation

The annotation stage assigned a sentiment class to each of the 7672 cleaned tweets. The labelling was done by three annotators with a background in psychology, referred to here as A1, A2, and A3. A psychology background was chosen because the task is the recognition of an attitude expressed in short informal text, which is a judgement about the stance of the writer and not a valuation of the company. None of the three annotators works as a financial analyst. To cover the domain specific vocabulary, a glossary of Indonesian stock trading terms that occur in the tweets, such as “Auto Reject *Atas* (ARA)/Upper Auto Rejection Limit”, “Auto Reject *Bawah* (ARB)/Lower Auto Rejection Limit”, “Full Call Auction (FCA)/a trading mechanism applied to stocks under special monitoring”, “*cuan*/profit”, and “*nyangkut*/holding a stock at a loss”, was prepared and given to the annotators together with the criteria in Table 9.

**Table 9:** Sentiment class annotation criteria.

Sentiment Class	Tweet Characteristics	Tweet Example
Positive	States optimism or confidence about the stock, predicts a price increase, reports a gain, or praises company performance or a corporate action	*Saham KARW Gokil ARA terus dari harga 50 perak sampai 5900* ***English translation:****KARW stock is wild, it keeps hitting the upper auto rejection limit, from 50 rupiah up to 5900*
Negative	States pessimism, disappointment, or criticism, predicts a price decline, warns other investors, or reports a loss	*Yg beli KARW di pucuk lgi ketar ketir* ***English translation:****Those who bought KARW at the top are getting nervous*
Neutral	Discusses the stock or the company but does not express optimism or pessimism, for example a report of information, a question, or an analysis with no directional view.	*Gandeng PT Meratus Line Pemkot Parepare Bakal Kembangkan Industri Kargo* ***English translation:****The Parepare City Government Partners with PT Meratus Line to Develop the Cargo Industry*
Irrelevant	Returned by the ticker query but does not state a position on the stock. This covers four cases: news headlines or factual reports republished without comment, tweets written in a language other than Indonesian, advertisements and account promotions, and tweets in which the ticker string is used as an ordinary Indonesian word instead of a company reference	*Untuk kamu yang mau memahami cara dan strategi dasar trading dan investasi saham bisa cek di sini ya BBRI PTRO PANI ANTM MLPL* ***English translation:****For anyone who wants to understand the basics of stock trading and investing, it can be checked here, BBRI PTRO PANI ANTM MLPL (an account promotion in which the tickers are used only as a reach tag)*

Before the annotation, a briefing session was held in which the four classes, the glossary, and a set of example tweets were discussed, so that the three annotators applied the criteria in the same way. The main annotation was carried out in Label Studio Community Edition (version 1.23). Each of the three annotators was assigned the full set of 7672 tweets and worked independently, without access to the labels of the other annotators.

Each tweet was assigned to one of four classes: positive, negative, neutral, and irrelevant as shown in Table 9. The irrelevant class was included so that posts mentioning a ticker without expressing any view on the stock could be separated from the sentiment classes. Tweets labelled as irrelevant are retained in the released Tweet Data - ID.csv and Tweet Data - EN.csv together with their label, so that researchers can decide whether to include them. These tweets are excluded from the daily aggregation and therefore do not appear in Combined Data.csv, so a study that needs the irrelevant class has to work from the Tweet Data files.

Inter-annotator agreement was measured with Cohen’s Kappa for each pair of annotators and with Fleiss’ Kappa for the three annotators collectively, as shown in Table 10. The Kappa score between annotator A1 and annotator A2 was 0.9925, between annotator A1 and annotator A3 was 0.8256, and between annotator A2 and annotator A3 was 0.8238. The average agreement among the three annotators based on Fleiss’ Kappa was 0.8811. These values indicate strong agreement among the annotators in assigning sentiment categories. Disagreement occurred most often on tweets containing terms that carry more than one meaning. The string “AUTO” is the ticker of PT Astra Otoparts Tbk and is also an Indonesian word meaning “automatically”, and the word “*cuan*” is an Indonesian term for profit. These tweets were labelled based on the surrounding context.

**Table 10:** Inter-annotator agreement scores among three annotators.

Pair	Score
A1 – A2 Cohen’s Kappa	0.9925
A1 – A3 Cohen’s Kappa	0.8256
A2 – A3 Cohen’s Kappa	0.8238
Annotators (A = 3) Fleiss’ Kappa	0.8811

The final label of each tweet was determined by majority voting. All three annotators assigned the same label to 6751 tweets (88.00%), two of the three agreed on 917 tweets (11.95%), and all three assigned different labels to 4 tweets (0.05%). For those 4 tweets, a designated annotator who was the most experienced of the three in text annotation work conducted a final review to determine the conclusive label, and the review assigned 3 of them to the positive class and 1 to the negative class. The resulting distribution is 3490 neutral tweets (45.49%), 1753 positive tweets (22.85%), 1751 irrelevant tweets (22.82%), and 678 negative tweets (8.84%). The individual label of each annotator is released together with the final label in Annotation Matrix.csv. The overall label distribution is shown in Fig. 4, and the distribution for each stock is shown in Fig. 5 and Table 11.

**Fig. 4:**
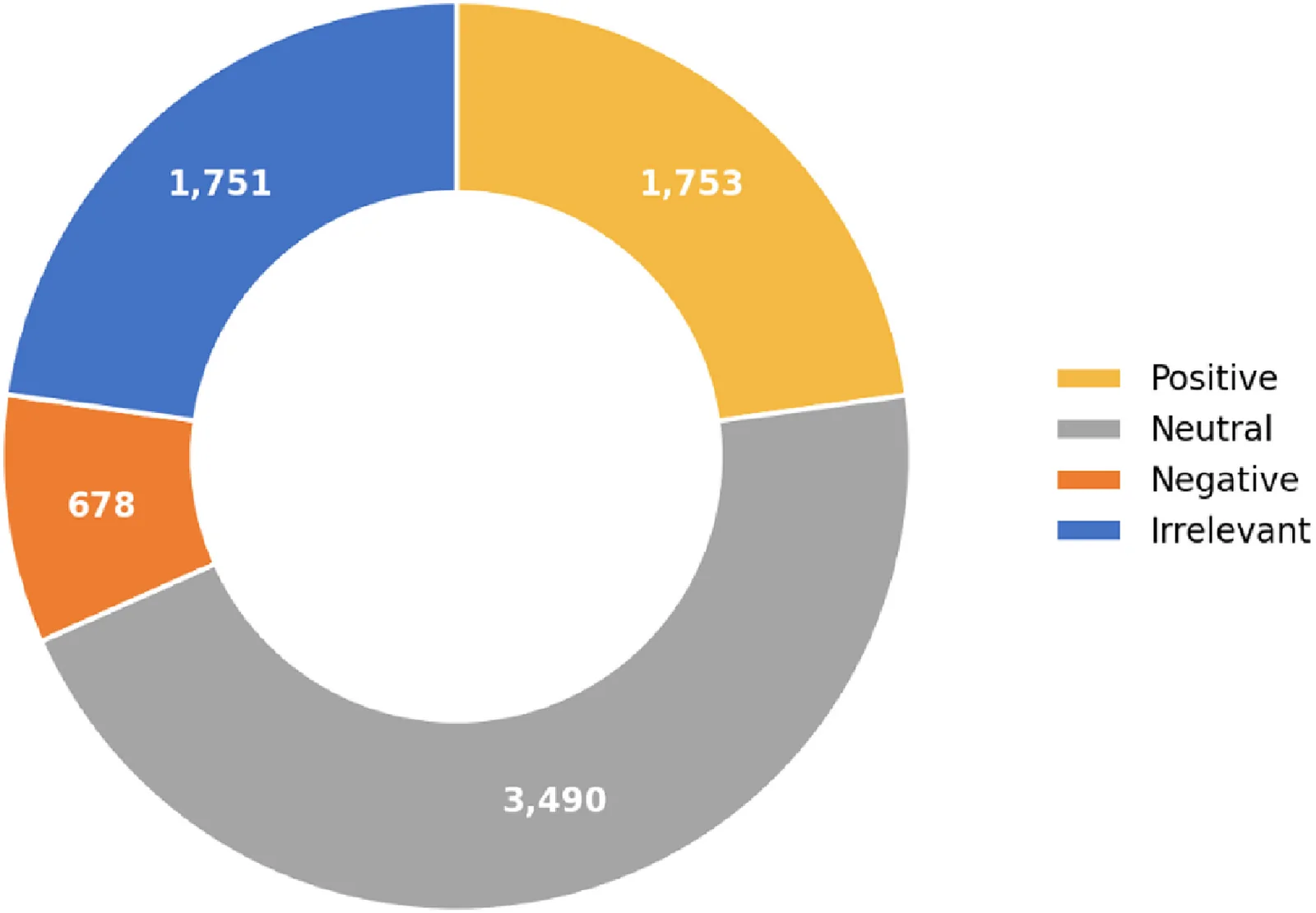
Label distribution for overall tweet.

**Fig. 5:**
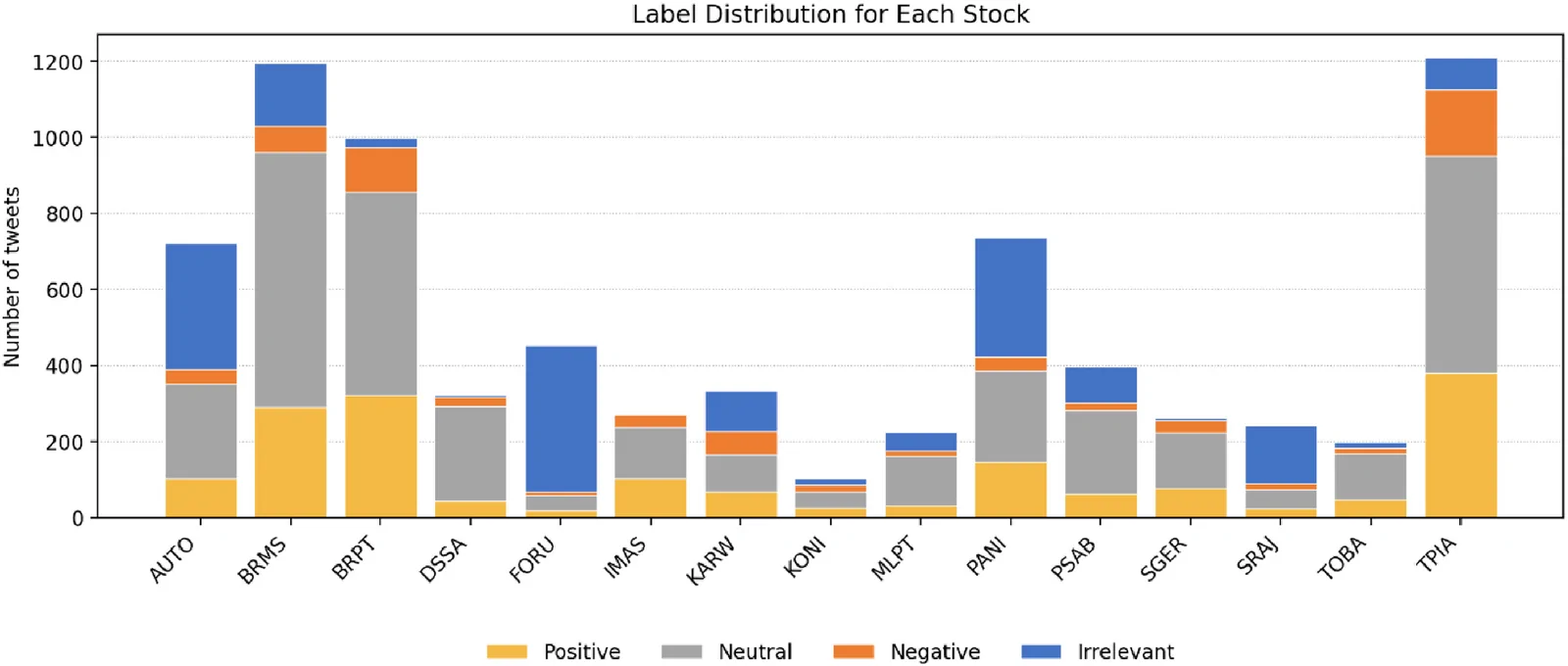
Label distribution for each stock.

**Table 11:** Label count of each stock.

Ticker	Positive	Neutral	Negative	Irrelevant
AUTO	103	249	39	330
BRMS	291	669	70	164
BRPT	324	533	116	23
DSSA	43	250	25	6
FORU	19	40	10	384
IMAS	104	134	33	3
KARW	68	98	62	104
KONI	26	42	17	18
MLPT	33	129	14	50
PANI	148	239	36	314
PSAB	63	220	19	96
SGER	79	145	32	6
SRAJ	24	51	15	153
TOBA	48	121	15	15
TPIA	380	570	175	85

### 4.7 Daily aggregation

The tweets with their final sentiment labels were then used for daily aggregation. Each label was converted into a numeric value, where positive was set to +1, neutral was set to 0, and negative was set to −1. Tweets labelled as irrelevant were excluded from the aggregation because they do not express a position on the stock. Tweets were grouped by ticker and by posting date in Western Indonesia Time. When several tweets fall on the same date for the same stock, their values were averaged into a single Daily Sentiment Score, which ranges from −1.0 (very negative) to +1.0 (very positive). The number of daily posts, also counted without the irrelevant tweets, was used to generate a uniform daily tweet count variable. The overall distribution of these sentiment scores is shown in Fig. 6.

**Fig. 6:**
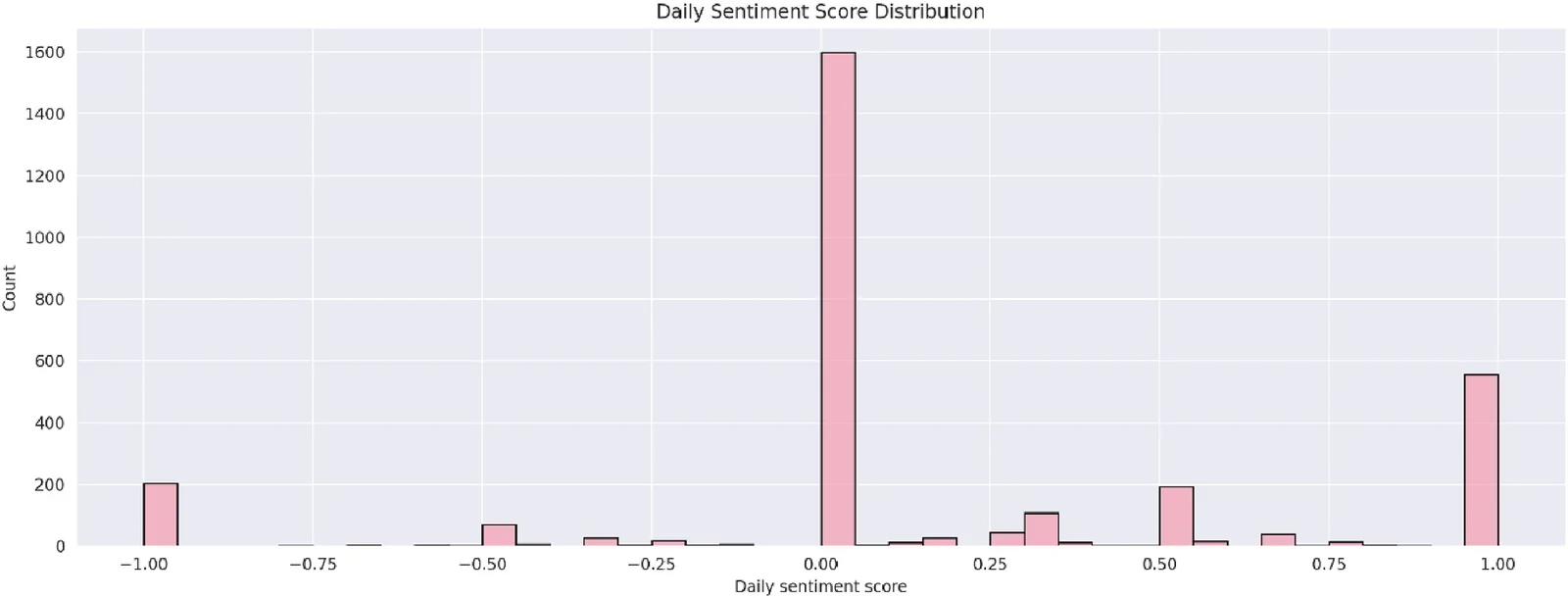
Daily sentiment score distribution.

The Tweet Data files also carry a trading_session_date column. A tweet posted on a trading day before the end of trading on that day is assigned to that trading day. The end of trading is taken as 15:00 Western Indonesia Time up to 31 March 2023, when orders for the closing price could be entered until 15:00 [11], and as 16:00 from 3 April 2023, when the same pre-closing order entry ends at 16:00 [16]. Every other tweet, including one posted later in the day, on a weekend, or on a public holiday, is assigned to the next trading date of the same ticker in the Market Data. The column is left empty for tweets posted after the last trading session of the observation period. The daily aggregation described above is based on the calendar posting date, while a study that requires no tweet to be linked to a price set before it was posted can group the tweets by trading_session_date and join the result to the market data on that column.

### 4.8 Data merging

The last stage in the construction of the dataset is the combination of the tweet and market data. The integration was performed via a left-join using the explicit common keys. The daily market variables were combined with the daily sentiment variables using the columns “ticker” and “date” as the primary join keys. The resulting file was named “Combined Data.csv”. This document contains the daily market data (the daily open, high, low, close, daily volume, daily RSI, daily percentage change in price, daily percentage change in volume), the daily tweet count, and the daily average sentiment score. The Combined Data contains a total of 16,395 rows. Tweets labelled as irrelevant were excluded before this stage, so the Combined Data summarises 5919 of the 5921 tweets that state a position on the stock. The remaining two were posted after the last trading session of the observation period. In cases where there were no tweets for a given ticker on a particular day, the values of the two fields tweet_count and daily_avg_score were set to 0.

To merge the tweets with the market data, only the date component of the created_at timestamp was used. Tweets posted with a timestamp on the same calendar day were assigned the same date. Tweets posted outside IDX trading hours and on weekends or public holidays were also assigned their own calendar date. Studies that need an alignment in which no tweet is linked to a price that was set before the tweet was posted can use the trading_session_date column described in Section 4.7. Because the raw tweets data had already been summarized into daily tweet summaries, the merging process required only the daily summarized data. Daily tweet metrics were summarized into the daily tweet count and the average daily sentiment score. This ensures the Combined Data is consistent and represents one calendar day for the one stock.

## Limitations

This dataset is limited to 15 non-blue-chip stocks listed on the Indonesia Stock Exchange and covers the period from 1 January 2022 to 31 December 2024. Tweet collection was based on stock-specific keywords, such as ticker symbols and company names. As a result, relevant discussions that do not contain these keywords may not be captured. The dataset is also limited to X platform, and discussions from other social media platforms and online forums are not represented. The use of targeted keyword queries may also introduce sampling bias, as the dataset does not capture all stock-related discussions on the platform.

The 15 stocks were selected for speculative price and volume behaviour, so patterns observed in this dataset are most applicable to stocks with similar trading characteristics and may differ for large-capitalisation stocks. The price to earnings ratio is provided in Stock Screening Values.csv as a supporting indicator of valuation, so studies that require a stricter valuation criterion can filter the stocks using these values. The English translation was reviewed manually after machine translation, and the Indonesian file remains the reference text for analyses that depend on exact wording.

Missing market data for non-trading days were handled using forward filling, in which the last observed market values were carried forward to subsequent non-trading dates. These imputed values do not represent new market observations and may not capture events or changes in sentiment occurring between trading sessions. The resulting Combined Data contains 16,395 calendar-aligned daily rows, of which 5565 (33.94%) repeat the values of the previous trading day. Consequently, price_change_pct and volume_change_pct are zero, and rsi_14 retains its previous value on these imputed rows. Users should therefore distinguish imputed rows from observed trading-day records when conducting analyses of market variability. These rows are marked by the is_imputed column in Combined Data.csv, so studies that require only observed market data can exclude them directly. This applies to trading volume as well, because the volume on a forward-filled day is copied from the previous trading day and does not reflect actual trading.

The sentiment labels may also be affected by automated or coordinated accounts, as bot activity was not explicitly identified or excluded during data collection. Manual annotation may be less reliable for tweets containing sarcasm, code-mixed text, or informal slang. In addition, tweets may be posted at any time during the day. Daily sentiment scores are therefore based on calendar dates and may include tweets posted after the market has closed. When the score is used to explain or predict the closing price of the same day, the tweets can be grouped by the trading_session_date column so that no tweet is linked to a price set before it was posted. These factors should be considered when interpreting relationships between daily sentiment and market variables. A tweet that mentions more than one of the selected tickers appears only once in the dataset and is linked to a single ticker, so the daily tweet count of a ticker may not include every tweet that mentions it.

The label distribution is imbalanced. Of the 7672 annotated tweets, 3490 are neutral (45.49%), 1753 are positive (22.85%), 1751 are irrelevant (22.82%), and 678 are negative (8.84%). Among the 5921 tweets that state a position on the stock, the neutral class is approximately five times larger than the negative class, and the class distribution also varies across tickers, as shown in Table 11. These characteristics may affect classification performance, particularly for the minority negative class. Users applying the dataset for classification should therefore consider class weighting, resampling, or threshold adjustment and report macro-averaged precision, recall, and F1 alongside accuracy. The dominance of the neutral class also means that daily_avg_score represents the net direction of stated opinion rather than discussion intensity. Therefore, tweet_count should be considered alongside daily_avg_score.

## Ethics Statement

All data were obtained from publicly available sources. The released text files contain the cleaned tweet text, the posting time, the aggregate engagement counts, and the assigned label. Account names, user mentions, user identifiers, post identifiers, and links to the original posts were removed before the files were released, so that a record cannot be traced back to its author or to the original post. The X Developer Policy [17] provides for X content to be shared in datasets as post and user identifiers, which would allow each post and its author to be retrieved. The posts are therefore released as de-identified text for non-commercial research, and any post will be removed from the repository upon request of its author. Consent was obtained from all annotators who participated in the data annotation stage. Market data were retrieved using the yfinance library from publicly available historical data provided by Yahoo Finance and were used for non-commercial academic research in accordance with the applicable terms of service [18].

## CRediT Author Statement

**Michael Sanjaya**: Conceptualization, Methodology, Software, Data Curation, Writing - Original Draft. **Kuncahyo Setyo Nugroho**: Methodology, Validation, Writing - Review & Editing. **Matthew Martianus Henry:** Methodology, Validation, Writing - Review & Editing**. Vera Intanie Dewi**: Supervision, Validation, Writing - Review & Editing. **Bens Pardamean**: Supervision, Validation, Writing - Review & Editing.

## Declaration of Competing Interests

The authors declare that they have no known competing financial interests or personal relationships that could have appeared to influence the work reported in this paper.

## Data Availability

Mendeley DataIndonesian Non-Blue-Chip Stocks: A Public Dataset of Daily Market Data and Tweets (Original data).
